# Componential Profile and Amylase Inhibiting Activity of Phenolic Compounds from *Calendula officinalis* L. Leaves

**DOI:** 10.1155/2014/654193

**Published:** 2014-02-09

**Authors:** Daniil N. Olennikov, Nina I. Kashchenko

**Affiliations:** Laboratory of Medical and Biological Research, Institute of General and Experimental Biology, Siberian Division, Russian Academy of Science, Sakh'yanovoy Street, 6, Ulan-Ude 670047, Russia

## Abstract

An ethanolic extract and its ethyl acetate-soluble fraction from leaves of *Calendula officinalis* L. (Asteraceae) were found to show an inhibitory effect on amylase. From the crude extract fractions, one new phenolic acid glucoside, 6′-*O*-vanilloyl-**β**-D-glucopyranose, was isolated, together with twenty-four known compounds including five phenolic acid glucosides, five phenylpropanoids, five coumarins, and nine flavonoids. Their structures were elucidated based on chemical and spectral data. The main components, isoquercitrin, isorhamnetin-3-*O*-**β**-D-glucopyranoside, 3,5-di-*O*-caffeoylquinic acid, and quercetin-3-*O*-(6′′-acetyl)-**β**-D-glucopyranoside, exhibited potent inhibitory effects on amylase.

## 1. Introduction

The Compositae annual herbaceous plant, *Calendula officinalis* L., commonly called *marigold*, or *pot marigold* is widely cultivated as an ornamental, culinary and valuable medicinal herb due to its various pharmacological properties: anti-inflammatory, antioxidant, antibacterial, antifungal, and so forth [1]. A number of chemical investigations have revealed the presence of several classes of compounds, with the main ones being terpenes, flavonoids, carotenoids, lipids, and carbohydrates.

Despite the wide use of *C. officinalis* flowers, leaves of this species have not currently found a practical application. The productivity of the vegetative foliage is much greater than that of the flowers, making it possible to consider the foliage as a new kind of useful plant material. The presence of various classes of compounds in *C. officinalis* leaves was determined as a result of chemical investigations. Isorhamnetin, isorhamnetin-3-*O*-*β*-d-glucopyranoside, and narcissin [2] belong to the phenolic compounds. Previously it has been shown that phenolic compounds are responsible for the presence of antioxidant and anticholinesterase activity in extracts of *C. officinalis* [3]. Carotenoids represent 12 compounds, dominated by lutein, *β*-carotene, and violaxanthin; the total carotenoid content in the leaves of *C. officinalis* growing in Bulgaria reaches 0.85 mg/g [4]. The most investigated groups of compounds found in the leaves of *C. officinalis* are triterpene glycosides and sterols. In young leaves the presence of cholestanol, campestanol, stigmastanol, and clerosterol derivatives in free, esterified, and glycosylated forms was revealed [5]. Mono-, di-, tri-, and tetraglucosides of olenolic acid isolated from *C. officinalis* leaves growing in Poland demonstrated antibacterial and antiparasitic activity [6]. The essential oil of *C. officinalis* leaves contains a set of compounds, dominated by sesquiterpenes (*τ*-muurolol, *δ*-cadinene) and monoterpenes (*α*-thujene) [7]. A number of neutral components, phospho-, and glycolipids have also been identified in the lipid complex [8].

In our previous investigation we detected some phenylpropanoids (caffeic acid, mono- and di-*O*-caffeoylquinic acids) and flavonoids (calendoflavoside, isoquercitrin, quercetin-3-*O*-(6′′-acetyl)-*β*-d-glucopyranoside, isorhamnetin-3-*O*-*β*-d-glucopyranoside, and isorhamnetin-3-*O*-(6′′-acetyl)-*β*-d-glucopyranoside) in *C. officinalis *leaves [9]. That work was realized just for “Orange Big” variety which is one of the most frequently cultivated double-flowered varieties in Russia. However, a number of *C. officinalis *varieties cultivated in Russia are much more indicating the necessity in extended chemical surveys.

Early, Yoshikawa et al. revealed a high hypoglycemic activity of the extracts and individual compounds from *C. officinalis* flowers that can be considered marigold as a forthcoming antidiabetic remedy [10]. In the course of our studies on the bioactivity of *C. officinalis* we found that an ethanolic extract from the leaves of this plant species showed inhibitory effect on the amylase. It is known that inhibitors of amylase, a carbohydrate hydrolyzing enzyme in the small intestine, are relevant to type II diabetes [11].

In this study, we present the results of phytochemical investigation of *C. officinalis* leaves from nine double-flowered varieties growing in the Russian Federation. As a result, twenty-five compounds were isolated including a new glycoside, 6′-*O*-vanilloyl-*β*-d-glucopyranose. In addition, we describe the inhibitory effects of the isolated compounds on amylase.

## 2. Materials and Methods

### 2.1. Plant Material

Plants of *Calendula officinalis *L. in nine double-flowered varieties (“Egypt Sun,” “Flame Dance,” “Geisha,” “Green Heart Orange,” “Indian Prince,” “Radio,” “Red Black Centered,” “Russian Size,” “Touch of Red”) were grown from authenticated seeds obtained from Tsitsin's Main Botanical Garden of the Russian Academy of Science (Moscow, Russian Federation) by cultivation in the fields of the Botanical Garden of the Institute of General and Experimental Biology (IGEB, Ulan-Ude, Russian Federation). The leaves were collected in the middle of August, 2012, and then dried *in vacuo* at 40°C (12 h) and stored at 4°C in the IGEB Plant Repository.

### 2.2. General Experimental Procedures

Elemental composition was determined using a MAT 8200 spectrometer (Thermo Finnigan). UV spectra were recorded using a SF-2000 spectrophotometer (OKB Specter). MS spectra were registered on a LCQ mass spectrometer (Thermo Finnigan). NMR spectra were recorded on a VXR 500S spectrometer (Varian). Chromatography was performed over columns of silica gel 60 (NP-SiO_2_; 230–400 mesh, Merck), Sephadex LH-20 (25–100 **μ**m, Pharmacia), polyamide (Woelm), octadecyl-functionalized silica gel (RP-SiO_2_; Aldrich), and Amberlite XAD7HP (Sigma). pTLC was performed on Sorbfil-A silica gel TLC plates (layer thickness 2 mm; Imid Ltd.). All chemicals were analytical grade. Alkaline hydrolysis [9], acidic hydrolysis [12], and HPLC analyses of cleavage products [13] were performed as described previously. The Folin method was used to determine total phenolic content (TPC) as described by [14] using chlorogenic acid as a standard compound. Total flavonoid content (TFC) was determined by a spectrophotometric method [15] using isoquercitrin as a standard compound.

### 2.3. Extraction and Isolation

Air-dried, ground leaves of *C. officinalis* (1.65 kg) were extracted three times with 60% EtOH at 80°C and the extracts were concentrated under reduced pressure to yield 462.7 g of crude extract. The crude extract was resuspended in water (1 : 6, *v*/*v*) and successively partitioned with hexane, CHCl_3_, EtOAc, and *n*-BuOH. The organic layers were brought to dryness *in vacuo *to yield 49.5, 79.4, 59.2, and 204.6 g of hexane (F1), CHCl_3_ (F2), EtOAc (F3), and *n*-BuOH fraction (F4) residue, respectively. The F2 fraction (54 g) was chromatographed over a Sephadex LH-20 column (6 × 100 cm) and eluted with CHCl_3_-MeOH (100 : 0→0 : 100) to obtain 10 fractions (frs. F2/1–F2/10). Frs. F2/2-F2/3 were combined and chromatographed on a silica column (3 × 50 cm) eluted with CHCl_3_-MeOH (100 : 0→70 : 30) to obtain 10 fractions (frs. F2/2-3/1–F2/2-3/10), which were separated using pTLC (solvent-toluene-EtOAc-HCOOH 6 : 3 : 1) to give umbelliferone (**12**; 6 mg), aesculetin (**13**; 14 mg) and scopoletin (**16**; 5 mg) [16]. Frs. F2/5-F2/7 were combined and chromatographed on a silica column (2.5 × 70 cm) and eluted with CHCl_3_-MeOH (100 : 0→70 : 30) to obtain quercetin (**17**; 19 mg) and isorhamnetin (**21**; 9 mg) [17]. Fr. F3 (50 g) was subjected to chromatography on a XAD7HP column (500 g) eluted with H_2_O (8 L), 40% EtOH (12 L) and 90% EtOH (10 L). The eluates were brought to dryness *in vacuo *to yield 1.3, 37.9, and 6.4 g of H_2_O (F3-1), 40% EtOH (F3-2) and 90% EtOH fraction (F3-3) residue, respectively. Fr. F3-2 was chromatographed on a polyamide column (5 × 120 cm) eluted with H_2_O-MeOH (100 : 0→0 : 100) to obtain 10 fractions (fr. F3-2/1–fr. F3-2/10). Frs. F3-2/2–F3-2/3 were separated on a Sephadex LH-20 column (4 × 140 cm) and eluted with MeOH-H_2_O (100 : 0→0 : 100) to obtain caffeic acid (**7**; 24 mg) [18], 3,5-di-*O*- (**9**; 184 mg), 1,5-di-*O*- (**10**; 62 mg) and 4,5-di-*O*-caffeoylquinic acid (**11**; 37 mg) [19]. Frs. F3-2/4–F3-2/7 were separated on a RP-SiO_2_ column (4 × 140 cm) and eluted with H_2_O-MeCN (100 : 0→0 : 100) to give 3-*O*-caffeoylquinic acid (**11**; 215 mg) [18], aesculin (**14**; 28 mg), cichoriin (**15**; 14 mg) [20], isoquercitrin (**18**; 182 mg), and isorhamnetin-3-*O*-*β*-d-glucopyranoside (**22**; 63 mg) [21]. Fr. F3-3 was subjected to pHPLC chromatography [Summit HPLC-system with a UV-Vis detector (Dionex) using a LiChrosorb RP-18 column (4.6 × 250 mm, 5 *μ*m, Merck), T 35°C, flow rate 1 mL min^−1^, solvent a linear gradient of 5–40% MeCN in H_2_O for 90 min, the detector at 360 nm; five runs] to give quercetin-3-*O*-(6′′-acetyl)-*β*-d-glucopyranoside (**19**; 54 mg) [22] and isorhamnetin-3-*O*-(6′′-acetyl)-*β*-d-glucopyranoside (**23**; 25 mg) [9]. Fr. F4 (120 g) was added to a XAD7HP column (800 g) and eluted with H_2_O (24 L), 40% EtOH (28 L) and 90% EtOH (5 L). The eluates were brought to dryness *in vacuo *to yield 54.9, 38.4, and 12.5 g of H_2_O (F4-1), 40% EtOH (F4-2), and 90% EtOH fraction (F4-3) residue, respectively. Fr. F4-2 was chromatographed on a polyamide column (5 × 120 cm) and eluted with H_2_O-MeOH (100 : 0→0 : 100) to give 10 fractions (F4-2/1–F4-2/10). Frs. F4-2/1–F4-2/2 were separated via pHPLC [Summit HPLC-system with UV-Vis detector (Dionex), LiChrosorb RP-18 column (4.6 × 250 mm, 5 *μ*m, Merck), T 35°C, flow rate 1 mL min^−1^, solvent a linear gradient of 0–10% MeCN in H_2_O for 90 min, the detector at 280 nm; four runs] to give 1′-*O*-*p*-hydroxybenzoyl-*β*-d-glucopyranose (**1**; 24 mg) [23], 6′-*O*-*p*-hydroxybenzoyl-*β*-d-glucopyranose (**2**; 11 mg), 1′-*O*-protocatechuoyl-*β*-d-glucopyranose (**3**; 18 mg), 6′-*O*-protocatechuoyl-*β*-d-glucopyranose (**4**; 14 mg) [24], 1′-*O*-vanilloyl-*β*-d-glucopyranose (**5**; 18 mg) [25], and compound **6** (27 mg). Frs. F4-2/4–F4-2/6 were separated on a Sephadex LH-20 column (4 × 140 cm) eluted with MeOH-H_2_O (100 : 0→0 : 100) to obtain rutin (**20**; 34 mg), narcissin (**24**; 24 mg) [21] and thyphaneoside (**25**; 23 mg) [26].

### 2.4. 6′-O-Vanilloyl-*β*-d-glucopyranose **(6)**: C_14_H_18_O_9_

UV *λ*_max⁡_ nm MeOH: 220, 261, 292. HR-ESI-MS, *m*/*z*: 353.2764 [M + Na]^+^ (calcd for C_14_H_18_O_9_Na 353.2840). +FAB-MS, *m*/*z*: 331 [M + H]^+^. ^1^H-NMR (CD_3_OD, 500 MHz) and ^13^C-NMR data (CD_3_OD, 125 MHz); see Table 2.

### 2.5. Sample Preparation and Analytical HPLC-UV

The dried and powdered leaves (200 mg) from the different varieties of *C. officinalis* were extracted with 60% ethanol (5 mL) in an ultrasonic bath for 40 min. The extracted solutions were filtered through a 0.22 *μ*m PTFE syringe filter before injection into the LC system for analysis.

HPLC analysis was performed on a MiLiChrom A-02 microcolumn chromatograph (Econova) coupled with a UV detector, using a ProntoSIL-120-5-C18 AQ column (2 × 75 mm, *∅* 5 *μ*m; Metrohm AG). Mobile phase A was 0.2 M LiClO_4_ in 0.006 M HClO_4_ and mobile phase B was acetonitrile. The injection volume was 1 *μ*L, and elution was at 150 *μ*L min^−1^ with a gradient program (0–7.5 min 11–18% B, 7.5–13.5 min 18% B, 13.5–15 min 18–20% B, 15–18 min 20–25% B, 18–24 min 25% B, and 24–30 min 25–100% B). The detector wavelength was 270 nm. Reference compounds with purity greater than 96% were used for the establishment of calibration curves. This included 3-*O*-caffeoylquinic acid, 3,5-di-*O*-caffeoylquinic acid, 1,5-di-*O*-caffeoylquinic acid, caffeic acid, isoquercitrin, and rutin from Sigma-Aldrich (Missouri, USA); Isorhamnetin-3-*O*-*β*-d-glucoside was from Extrasynthese (Lyon, France); 4,5-di-*O*-caffeoylquinic acid was from ChemFaces (Wuhan, China). Thyphaneoside, quercetin-3-*O*-(6′′-acetyl)-*β*-d-glycoside, and isorhamnetin-3-*O*-(6′′-acetyl)-*β*-d-glycoside were isolated previously from *C. officinalis* flowers [9].

### 2.6. Amylase Inhibition Microplate Assay

Amylase inhibitory activity was measured using a microplate method: 10 *μ*L of a sample solution in DMSO, 30 *μ*L of phosphate buffer (pH 5.0), and 10 *μ*L of amylase from *Aspergillus niger* (3 U mL^−1^, Sigma) which were incubated for 20 min at 45°C. Then 10 *μ*L of 2% starch solution, 40 *μ*L of phosphate buffer (pH 5.0), and 100 *μ*L of the reagent were added and incubated for 30 min at 50°C. Absorbance was measured at 510 nm. The reagent was a solution of K_2_HPO_4_ (0.8 mM), KH_2_PO_4_ (0.4 mM), phenol (220 mM), 4-aminoantipyrine (1.5 *μ*M), glucose oxidase from *Aspergillus niger* (3 U mL^−1^; Sigma), and peroxidase from horseradish (0.3 U mL^−1^) in deionized water. A 2% solution of acarbose was used as a positive control (PC), and water was used as a negative control (NC). The experiment was carried out in triplicate and averaged. The ability to inhibit amylase was calculated using the following equation: inhibitory ability (%) = [(A_510_^NC^ − A_510_^PC^) − (A_510_^Sample^ − A_510_^PC^)/(A_510_^NC^ − A_510_^PC^)] × 100, where A_510_^NC^ is the absorbance of the negative control, A_510_^PC^ is the absorbance of the positive control, and A_510_^Sample^ is the absorbance of the sample solution. The IC_50_ value is the effective concentration at which amylase activity was inhibited by 50%. Values are expressed as mean obtained from 5 independent experiments.

## 3. Results and Discussion

### 3.1. Phenolic Compounds Content in Russian Varieties of *C. officinalis* Leaves

**Figure 1:**
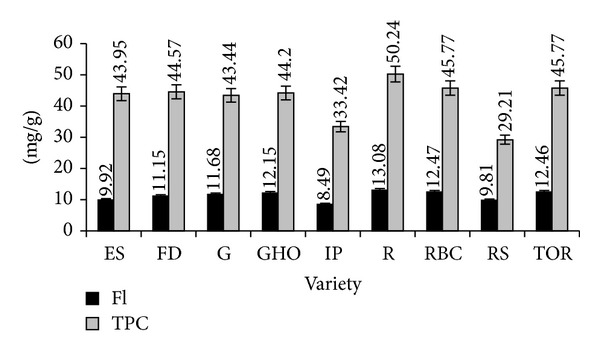
Total flavonoid content (black bars; Fl) and total phenolic content (grey bars; TPC) in nine varieties of *C. officinalis *leaves. Varieties: “Egypt Sun” (ES), “Flame Dance” (FD), “Geisha” (G), “Green Heart Orange” (GHO), “Indian Prince” (IP), “Radio” (R), “Red Black Centered” (RBC), “Russian Size” (RS), and “Touch of Red” (TOR). Under the bars—means of content, mg g^−1^.

Preliminary chemical research on the composition of *C. officinalis* leaves was carried out for nine varieties cultivated widely in the territory of Russia. These varieties are characterized by high productivity and simplicity of cultivation. As a result, it was found that flavonoid content in the leaves examined ranged from 8.49 (“Indian Prince”) to 13.08 mg g^−1^ (“Radio”), while the total content of phenolic compounds varied from 29.21 (“Russian Size”) to 50.24 mg g^−1^ (“Radio”) (Figure 1). The maximal content of phenolic compounds was observed for *C. officinalis* leaves of the “Radio” variety, which were further subjected to detailed chemical study.

### 3.2. Extraction and Isolation of Phenolic Compounds from *C. officinalis* Leaves of “Radio” Variety

A 60% ethanolic extract of *C. officinalis* leaves of “Radio” variety was partitioned with CHCl_3_, EtOAc, and *n*-BuOH to yield three fractions. The crude extract was found to exhibit inhibitory activity on an amylase with an inhibitory value (IC_50_) of 38.02 ± 1.29 **μ**g mL^−1^ (Table 1). In the same bioassay, the ethylacetate-soluble fraction still showed potent inhibitory activity with a lower value, 24.52 ± 0.88 **μ**g mL^−1^.

**Figure 2:**
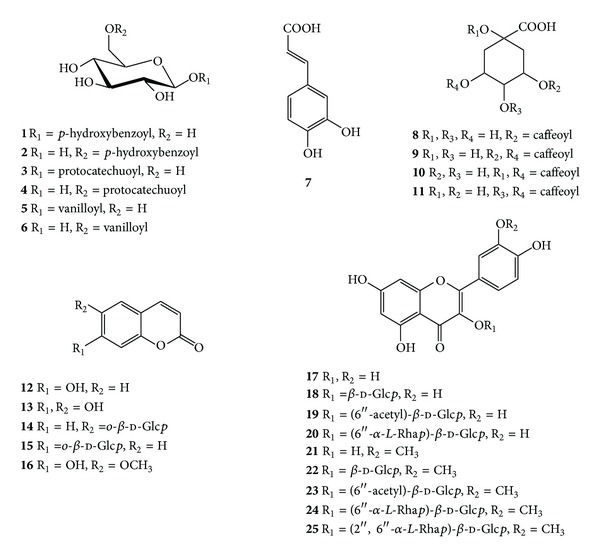
Chemical structures of compounds **1**–**25** isolated from *C. officinalis* leaves. Glc*p*—glucopyranose, Rha*p*—rhamnopyranose.

To enhance our knowledge regarding the chemical composition of *C. officinalis* leaves, all the isolated fractions were separated by chromatographic columns (gel permeation, NP- and RP-SiO_2_, XAD, and polyamide chromatography), prep. HPLC, and prep. TLC, yielding one new (**6**) and twenty-four known compounds. The known compounds, including five phenolic acid glucosides [1′-*O*-*p*-hydroxybenzoyl-*β*-d-glucopyranose (**1**), 6′-*O*-*p*-hydroxybenzoyl-*β*-d-glucopyranose (**2**), 1′-*O*-protocatechuoyl-*β*-d-glucopyranose (**3**), 6′-*O*-protocatechuoyl-*β*-d-glucopyranose (**4**), and 1′-*O*-vanilloyl-*β*-d-glucopyranose (**5**)], five phenylpropanoids [caffeic acid (**7**), 3-*O*- (**8**), 3,5-di-*O*- (**9**), 1,5-di-*O*- (**10**), and 4,5-di-*O*-caffeoylquinic acid (**11)**], five coumarins [umbelliferone (**12**), aesculetin (**13**), aesculin (**14**), cichoriin (**15**), scopoletin (**16)**], and nine flavonoids [quercetin (**17**), isoquercitrin (**18**), quercetin-3-*O*-(6′′-acetyl)-*β*-d-glucopyranoside (**19**), rutin (**20**), isorhamnetin (**21**), isorhamnetin-3-*O*-*β*-d-glucopyranoside (**22**), isorhamnetin-3-*O*-(6′′-acetyl)-*β*-d-glucopyranoside (**23**), narcissin (**24**), and thyphaneoside (**25)**], were identified by comparing their UV, MS, and NMR data with those reported in the literature (Figure 2).

Compounds **7**, **9**, **18**, **19**, and **21**–**24** have been previously isolated [2, 9], while compounds **1**–**5**, **8**, **10**–**17**, **20,** and **25** are detected in *C. officinalis* leaves for the first time.

### 3.3. Structure Elucidation of 6′-O-Vanilloyl-*β*-d-glucopyranose

Compound **6** was isolated as a white amorphous powder. Its molecular formula, C_14_H_18_O_9_, was deduced from its [M + H]^+^ peak at 331 of positive FAB-MS and the 14 carbon signals in its ^13^C-NMR spectrum. In its ^1^H-NMR spectrum the signals of three aromatic protons were observed at *δ* 7.63 (1H, dd, *J* = 8.0, 1.9 Hz), 7.52 (1H, d, *J* = 1.9 Hz), and 6.80 (1H, d, *J* = 8.0 Hz), which are typical for 1,3,4-trisubstituted benzene rings (Table 2).

**Figure 3:**
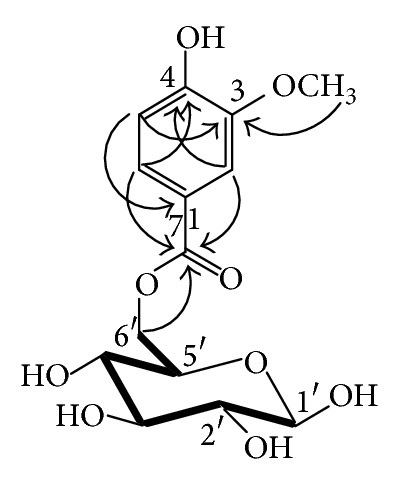
Selected ^1^H-^1^H COSY (**—**)correlations in HMBC spectrum (H→C) of **6**.

Its ^13^C-NMR spectrum exhibited a carbonyl group at *δ* 166.7 and a methoxyl group at *δ* 56.3. In its HMBC spectrum, the carbonyl group (*δ* 166.7) showed a correlation with the aromatic proton of H-6 at *δ* 7.63 and the aromatic carbon at *δ* 147.5 (C-3) correlated with methoxyl protons at *δ* 3.97 (Figure 3).

Alkaline hydrolysis of **6** with 1 M KOH gave vanillic acid, which was identified by *co*-HPLC with an authentic sample and ^13^C-NMR. These data suggest the presence of a vanilloyl moiety in the structure of **6**. The presence of d-glucose was confirmed after acidic hydrolysis followed by HPLC analysis of the hydrolysate. In addition, the remaining 6 carbon signals in its ^13^C-NMR spectrum are in good agreement with those for *β*-glucose. The linkage of the vanilloyl moiety and glucose was solved by an analysis of the HMBC spectrum. The carbonyl group (**δ**166.7) showed correlation with the proton at **δ**4.05, suggesting that the vanilloyl moiety is connected to C-6′ of the glucose moiety. Moreover, the results of the ^13^C-NMR analysis showed a downfield shift in the resonance of the glucose C-6′, as well as an upfield shift of the C-5′ resonance comparing with those of free glucose. Assignment of the protons and carbons signals was achieved by a combination of the HMBC and ^1^H-^1^H COSY spectral data. Since the anomeric hydroxyl group of the glucose moiety of **6** is unacylated, it may be obtained as anomers (*α*- and *β*-forms) mixture. The ^13^C-NMR data obtained showed a presence of a week signal at **δ**92.1 that corresponded to *α*-form of **6**. However, the ratio of the peak areas for C-1′ atoms of *α*- (**δ**92.1) and *β*-forms (**δ**98.3) is about 1 : 92. Thus, we can conclude that the predominant component of mixture is *β*-forms (total content of *α*-form is no more than 1.1%). These results enabled compound **6** to be identified as 6′-*O*-vanilloyl-*β*-d-glucopyranose.

The moiety of **6** has been detected previously in different compounds like glehlinosides from *Glehnia littoralis* (Euphorbiaceae) [27], 6′-*O*-vanilloylsucrose from *Saccharum officinarum *(Poaceae) [28], 6′-*O*-vanilloyicariside B_5_ from *Baccaurea ramiflora* (Euphorbiaceae) [29], amburoside G from *Amburana cearensis *(Fabaceae) [30], saccharumosides from *Acer saccharum* (Aceraceae) [31], and 1′-*O*-galloyl-6′-*O*-vanilloyl-*β*-d-glucopyranose from *Alchornea trewioides* (Euphorbiaceae) [32]. This is the first case where **6** has been isolated as a separate compound.

### 3.4. HPLC-UV Analysis of the Main Phenolic Compounds in *C. officinalis* Leaves

**Figure 4:**
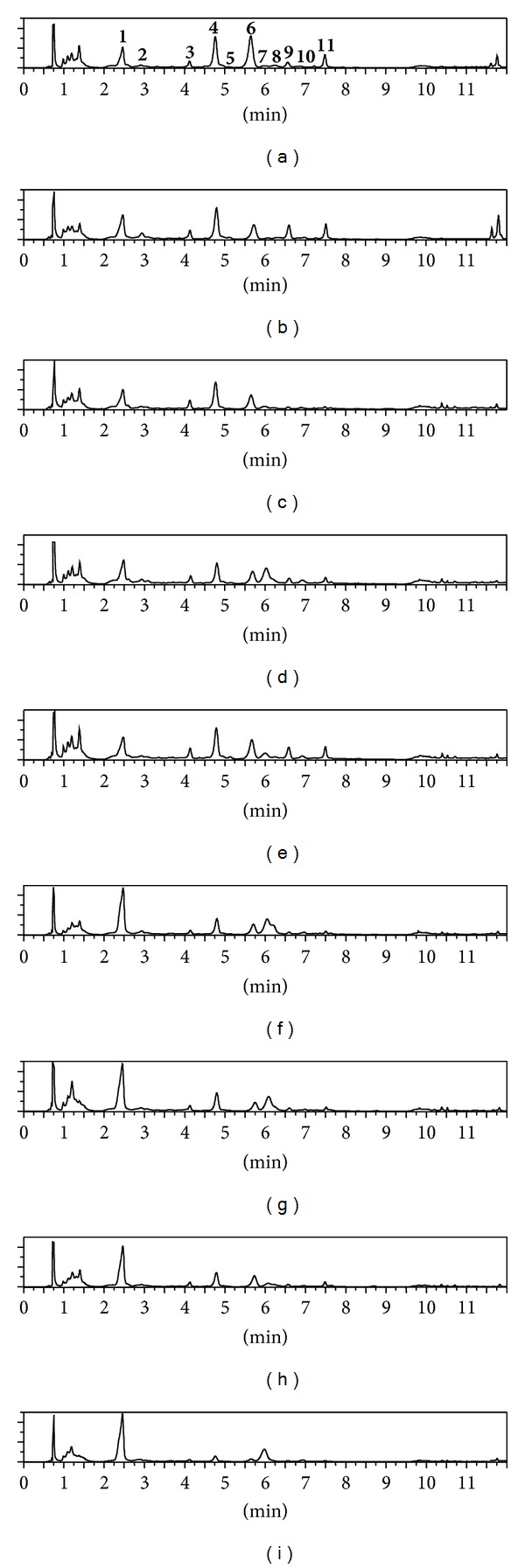
HPLC-UV chromatograms (270 nm) of ethanolic extracts from *C. officinalis *leaves. Varieties: (a)—“Touch of Red,” (b)—“Green Heart Orange,” (c)—“Russian Size,” (d)—“Indian Prince,” (e)—“Red Black Centered,” (f)—“Radio,” (g)—“Egypt Sun,” (h)—“Geisha,” (i)—“Flame Dance.” Compounds: **1**—3-*O*-caffeoylquinic acid, **2**—caffeic acid, **3**—thyphaneoside, **4**—isoquercitrin, **5**—rutin, **6**—quercetin-3-*O*-(6′′-acetyl)-*β*-d-glycoside, **7**—3,5-di-*O*-caffeoylquinic acid, **8**—1,5-di-*O*-caffeoylquinic acid, **9**—isorhamnetin-3-*O*-*β*-d-glucoside, **10**—4,5-di-*O*-caffeoylquinic acid, and **11**—isorhamnetin-3-*O*-(6′′-acetyl)-*β*-d-glycoside.

A quantitative analysis of the main phenolic compounds found in *C. officinalis *leaves was performed using microcolumn HPLC-UV method which allowed separating 15 components (Figure 4). This method was developed previously for analysis of *C. officinalis *flowers [9].

According to the HPLC data, isoquercitrin—3.44 (“Indian Prince”)—5.34 mg g^−1^ (“Green Heart Orange”, “Red Black Centered”) was the dominant compound, as well as 3-*O*-caffeoylquinic acid—6.48 (“*Egypt Sun*”)—13.25 mg g^−1^ (“Flame Dance”) (Table 3). The total content of phenylpropanoids ranged from 2.58 mg g^−1^ in “Touch of Red” to 20.17 mg g^−1^ in “Flame Dance.” 3-*O*-caffeoylquinic acid was a major phenylpropanoid component for all species studied. Monocaffeoylquinic acids in all varieties predominated over dicaffeoylquinic acids. The concentration of caffeic acid did not exceed 0.29–1.14 mg g^−1^. The total content of flavonoids varied from 6.11 mg g^−1^ in “Flame Dance” to 15.74 mg g^−1^ in “Touch of Red.”

The content of quercetin derivatives was higher than the content of derivatives of isorhamnetin, ranging from 1.64- (“Green Heart Orange”) to 4.79-fold (“Radio”). It should be noted that isoquercitrin dominated in almost all species except “Geisha” and “Touch of Red” where quercetin-3-O-(6′′-acetyl)-*β*-d-glycoside prevailed. Thyphaneoside in “Egypt Sun,” “Flame Dance,” “Indian Prince,” “Radio,” “Russian Size,” isorhamnetin-3-*O*-*β*-d-glucoside in “Green Heart Orange” and “Red Black Centered,” and isorhamnetin-3-*O*-(6′′-acetyl)-*β*-d-glycoside in “Geisha” and “Touch of Red” are all simple derivatives of isorhamnetin.

According to the data on the phenylpropanoids, flavonoid ratio, *C. officinalis* varieties studied can be divided into three groups:

1. varieties with a predominant content of flavonoids (value < 0.5)—“Touch of Red,” “Green Heart Orange,” “Red Black Centered” and “Russian Size”;
2. varieties with similar contents of phenylpropanoids and flavonoids (value 0.5–1.5)—“Radio,” “Egypt Sun,” “Geisha” and “Indian Prince”;
3. varieties with a predominance of phenylpropanoids (value > 1.5)—“Flame Dance.”

### 3.5. Amylase Inhibiting Activity of *C. officinalis* Phenolic Compounds

All substances isolated were tested for their amylase inhibiting activity by the microplate method. Compounds **7**, **13**, **17**, and **21 **showed significantly higher inhibitory activity at concentration ranging 1.02–2.64 *μ*g mL^−1^ comparing with the reference compound acarbose (9.54 **μ**g mL^−1^) (Table 4). It should be noted that concentrations of the mentioned compounds in *C. officinalis* leaves are insignificant; therefore, they cannot be considered as an active substances. Comparative analysis of the inhibitory activities of the dominant compounds showed that the actual antiamylase components in the crude extracts were **18** (isoquercitrin; 15.45 *μ*g mL^−1^), **22** (isorhamnetin-3-*O*-*β*-d-glucopyranoside; 23.41 *μ*g mL^−1^), **9** (3,5-di-*O*-caffeoylquinic acid; 25.63 *μ*g mL^−1^), and **19** (quercetin-3-*O*-(6′′-acetyl)-*β*-d-glucopyranoside; 32.70 *μ*g mL^−1^).

It is known that the phenolic phytosubstances are the perspective natural compounds which exhibited antidiabetic activity [33]. Flavonoids and phenylpropanoids demonstrate a high inhibitory activity on amylase and glucosidase, the key enzymes of carbohydrates digestion process [34]. Some caffeoylquinic acids (chlorogenic acid, isochlorogenic acid) and derivatives of quercetin and isorhamnetin, such as rutin (quercetin-3-*O*-rutinoside) and narcissin (isorhamnetin-3-*O*-rutinoside), have been previously reported as inhibitors of glucosidase [35]. The results of our experiments seemed to agree with this finding, probably due to the presence of large amounts of phenolic compounds in *C. officinalis* leaves.

The present investigation is the first extended study of the phenolic composition from *C. officinalis* leaves. As a result, twenty-five compounds were isolated, of which sixteen had not previously been reported in this plant source. The presence of undiscovered benzoic acid glucosides early in the *Calendula *genus, one of which was novel (6′-*O*-vanilloyl-*β*-d-glucopyranose), as well as the amylase inhibiting activity of the crude extract and individual compounds, illustrates the need for in-depth research even on very familiar species such as *C. officinalis*.

## Figures and Tables

**Table 1:** Amylase inhibiting activity of *C. officinalis* extract and fractions.

Sample	IC_50_ (*μ*g mL^−1^)
Ethanolic extract	38.02 ± 1.29
CHCl_3_ fraction	>100
EtAc fraction	24.52 ± 0.88
BuOH fraction	72.60 ± 2.61
Acarbose^a^	9.54 ± 0.32

Values are expressed as mean ± SD obtained from 5 independent experiments.

^a^Reference compounds.

**Table 2:** ^1^H-NMR and ^13^C-NMR data of compound **6**.

Atom no.	DEPT	*δ*_H_ (mult., *J* in Hz)	*δ*_*C*_
1	C		120.3
2	CH	7.52 (1H, d, 1.9)	112.7
3	C		147.5
4	C		153.4
5	CH	6.80 (1H, d, 8.0)	115.4
6	CH	7.63 (1H, dd, 8.0, 1.9)	125.6
7	C		166.7
4-OCH_3_	CH_3_	3.97 (3H, s)	56.3
1′	CH	4.56 (1H, d, 7.2)	98.3
2′	CH	3.39–3.62 (4H, m)	73.4
3′	CH		78.7
4′	CH		70.3
5′	CH		74.5
6′	CH_2_	4.37 (1H, dd, 11.9, 2.0) 4.05 (1H, dd, 11.9, 7.4)	64.2

**Table 3:** Content of phenolic compounds in nine varieties of *C. officinalis *leaves, mg g^−1^ from dry plant weight.

Compound	Variety								
	“Egypt Sun”	“Flame Dance”	“Geisha”	“Green Heart Orange”	“Indian Prince”	“Radio”	“Red Black Centered”	“Russian Size”	“Touch of Red”
Phenylpropanoids									

Caffeic acid	0.46	1.14	0.31	0.57	0.43	0.64	0.39	0.29	0.20
3-*O*-Caffeoylquinic acid	6.48	13.25	6.59	2.41	2.35	8.85	2.31	1.95	1.73
3,5-Di-*O*-caffeoylquinic acid	2.37	4.91	0.80	0.08	1.90	3.90	0.86	0.40	0.23
1,5-Di-*O*-caffeoylquinic acid	0.46	0.33	0.34	0.10	0.52	1.53	0.24	0.19	0.27
4,5-Di-*O*-caffeoylquinic acid	0.18	0.54	0.20	0.07	0.39	0.36	0.33	0.12	0.15
Total phenylpropanoids	**9.95**	**20.17**	**8.24**	**3.23**	**5.59**	**15.28**	**4.13**	**2.95**	**2.58**

Flavonoids									

Quercetin derivatives									
Isoquercitrin	3.93	2.73	4.32	5.34	3.44	5.02	5.34	4.59	5.14
Rutin	0.22	0.17	0.92	0.63	0.29	0.27	0.72	0.15	0.17
Quercetin-3-*O*-(6′′-acetyl)-*β*-d-glycoside	2.50	1.68	4.36	3.14	2.67	4.25	4.30	3.03	6.85
Total quercetin derivatives	**6.65**	**4.58**	**9.60**	**9.11**	**6.40**	**9.54**	**10.36**	**7.77**	**12.16**

Isorhamnetin derivatives									
Thyphaneoside	0.78	0.74	1.01	1.08	0.92	0.97	1.37	2.46	0.73
Isorhamnetin-3-*O*-*β*-d-glucoside	0.63	0.44	0.71	2.41	0.88	0.22	2.15	0.36	1.04
Isorhamnetin-3-*O*-(6′′-acetyl)-*β*-d-glycoside	0.62	0.35	1.21	2.08	0.81	0.80	1.64	0.32	1.81
Total isorhamnetin derivatives	**2.03**	**1.53**	**2.93**	**5.57**	**2.61**	**1.99**	**5.16**	**3.14**	**3.58**
Total flavonoids	**8.68**	**6.11**	**12.53**	**14.68**	**9.01**	**11.53**	**15.52**	**10.91**	**15.74**
Total identified	**18.63**	**26.28**	**20.77**	**17.91**	**14.60**	**26.81**	**19.65**	**13.86**	**18.32**
Quercetin/isorhamnetin ratio	3.28	2.99	3.28	1.64	2.45	4.79	2.01	2.48	3.40
Phenylpropanoid/flavonoid ratio	1.15	3.30	0.66	0.22	0.62	1.33	0.27	0.27	0.16

**Table 4:** Amylase inhibiting activity of individual phenolic compounds.

Compound	IC_50_ (*μ*g mL^−1^)
**1**	>100
**2**	>100
**3**	62.46
**4**	66.52
**5**	73.69
**6**	79.18
**7**	2.53
**8**	65.42
**9**	25.63
**10**	37.15
**11**	26.65
**12**	>100
**13**	1.02
**14**	>100
**15**	>100
**16**	~100
**17**	1.79
**18**	15.45
**19**	32.70
**20**	89.85
**21**	2.64
**22**	23.41
**23**	~100
**24**	>100
**25**	>100
Acarbose^a^	9.54

^a^Reference compounds.
